# Unprecedented carbonic anhydrase inhibition mechanism: Targeting histidine 64 side chain through a halogen bond

**DOI:** 10.1002/ardp.202400776

**Published:** 2025-01-06

**Authors:** Roberto Paciotti, Simone Carradori, Andrea Angeli, Ilaria D'Agostino, Marta Ferraroni, Cecilia Coletti, Claudiu T. Supuran

**Affiliations:** ^1^ Department of Pharmacy “G. d'Annunzio” University of Chieti‐Pescara Chieti Italy; ^2^ Section of Pharmaceutical and Nutraceutical Sciences, Department of Neuroscience, Psychology, Drug Research and Child Health (NEUROFARBA) University of Florence Sesto Fiorentino Firenze Italy; ^3^ Department of Pharmacy University of Pisa Pisa Italy; ^4^ Department of Chemistry “Ugo Schiff” University of Florence Sesto Fiorentino Florence Italy

**Keywords:** bithionol, carbonic anhydrase, fragment molecular orbital, halogen bond, kinetics

## Abstract

2,2′‐Thio‐bis(4,6‐dichlorophenol), namely bithionol, is a small molecule endowed with a multifaceted bioactivity. Its peculiar polychlorinated phenolic structure makes it a suitable candidate to explore its potentialities in establishing interaction patterns with enzymes of MedChem interest, such as the human carbonic anhydrase (hCA) metalloenzymes. Herein, bithionol was tested on a panel of specific hCAs through the stopped‐flow technique, showing a promising micromolar inhibitory activity for the hCA II isoform. X‐ray crystallographic studies revealed an unprecedented halogen‐bond interaction between one chlorine of bithionol and the N3(ε) atom of the hCA II catalytically active histidine residue, His64. Then, quantum mechanics calculations based on the fragment molecular orbital method allowed us to estimate the strength of this bond (~2.9 kcal/mol) and highlighted the contribution of a rich hydrophobic interaction network within the isoenzyme. Interestingly, the compound inactivity against the hCA III isoform, characterized by His64Lys and Leu198Phe mutations, supported the key role played by halogen bonding in the enzyme affinity. This finding might pave the way for the development of a new class of hCA inhibitors characterized by such chemical features, with the halogen bond being a key ligand–receptor interaction.

## Graphical abstract

The organochlorine bithionol showed a micromolar inhibition of specific human carbonic anhydrases (hCAs), and X‐ray crystallography revealed the presence of a halogen‐bond interaction between one chlorine and the N3 atom of the catalytically active His64 in hCA II. Fragment molecular orbital‐based quantum mechanics calculations were employed to gain insights into this interaction.

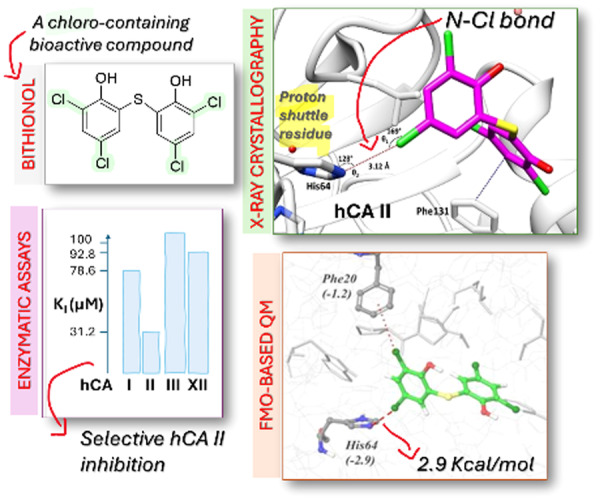

**Abbreviations**
AAZ: acetazolamide
ADME: absorption, distribution, metabolism, and excretion
CA: carbonic anhydrase
FMO: fragment molecular orbital
h: human
KI: inhibition constant
NCI: noncovalent interaction
PIEDA: pair interaction energy decomposition analysis
sAC: soluble adenylyl cyclase

## 1 INTRODUCTION

Protein complexes with halogenated ligands have attracted growing interest in recent years, presenting new opportunities for drug design.^[^ ^1^, ^2^, ^3^, ^4^ ^]^ Halogen elements, such as fluorine, chlorine, bromine, and iodine, are extensively studied in medicinal chemistry and integrated into drugs to enhance selectivity, improve absorption, distribution, metabolism, and excretion (ADME) properties, or reduce side reactions, making them a key strategy for developing more effective and selective therapeutic agents.^[^ ^5^, ^6^, ^7^ ^]^ Within this framework, nitrogen−halogen bonds represent favorable interactions and could prove valuable in designing modulators that interact with histidine residues in the active site of the proteins stabilizing protein–ligand complexes and influencing the overall binding affinity and specificity. These bonds, known as halogen bonds (X‐bonds), are attractive interactions between an electrophilic region of a halogen atom and a nucleophilic site on another molecular entity.^[^ ^8^ ^]^ The driving force behind this interaction is explained by the σ‐hole concept,^[^ ^9^, ^10^ ^]^ which describes how the halogens enhance their contacts with side‐chain oxygen and nitrogen atoms in the protein.^[^ ^11^ ^]^

This study aims to investigate the X‐bond effect in the modulation of promising pharmacological targets, such as carbonic anhydrase (CA) enzymes. The latter represents an important class of metalloenzymes that play a critical role in regulating the equilibrium between carbon dioxide (CO_2_) and bicarbonate (HCO_3_^–^) across all domains of life.^[^ ^12^, ^13^ ^]^ These enzymes are structurally classified into eight classes (α, β, γ, δ, ζ, η, θ, and ι), and, despite their different evolution, catalyze chemically equivalent transformations, underscoring their fundamental importance in cellular processes such as acid–base balance, ion transport, and pH regulation.^[^ ^14^, ^15^, ^16^, ^17^ ^]^ Therapeutically, the modulation of specific CA isoforms can be leveraged to treat a range of medical conditions, including glaucoma, epilepsy, hypoxic tumors, and, more recently, infectious diseases.^[^ ^18^, ^19^ ^]^

Although many structural and mechanistic studies have led to the discovery of several libraries of CA inhibitors, as far as our knowledge extends, no structure‐based molecular design has been developed targeting the rate‐limiting step of the CA mechanism where the proton shuttle residue, His64, is involved (residue numbering referred to human carbonic anhydrase [hCA] II).^[^ ^20^, ^21^, ^22^ ^]^ In this context, the X‐bond represents a novel possibility for developing new inhibitors that specifically target the proton shuttle residue.

**Figure 1:**
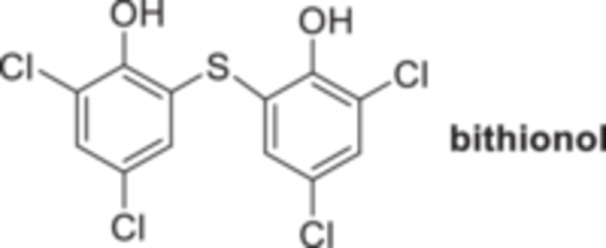
Chemical structure of bithionol.

Herein, we reported a case study involving the organochlorine compound bithionol (Figure 1) where its binding mode has been characterized by experimental studies and supported by computational simulations. Indeed, computational chemistry methods are widely used to discover and develop active molecules, allowing elucidating their binding modes with target receptors.^[^ ^23^ ^]^ In this work, we assessed the ability of bithionol to inhibit a focused panel of CAs and used quantum mechanics‐based methods to analyze and characterize the halogen bond established by bithionol and hCA II, rationalizing the anzymatic assays results.

## 2 RESULTS AND DISCUSSION

### 2.1 Rationale of the study

We hypothesized that its two OH moieties and Cl atoms could play a pivotal role in interacting with CA metalloenzymes through hydrophobic, ionic, and polar contacts. In fact, due to the physiopathological functions of CA enzymes, various inhibiting chemotypes have been explored in the last decade, mostly benzenesulfonamide and coumarins.^[^ ^24^, ^25^ ^]^ However, several phenolic and polyphenolic compounds have emerged for their ability to interact with CAs and modulate their activity.^[^ ^26^, ^27^ ^]^ In particular, their acidic hydroxyl group can form a hydrogen bond with the zinc‐bound water molecule, while the phenyl ring can generate hydrophobic interactions with apolar amino acid residues surrounding the active site.^[^ ^21^, ^26^, ^28^ ^]^ On the other hand, the introduction of halogen atoms has traditionally been explored in phenyl ring‐containing compounds, including CA inhibitors, with the specific aim of enhancing the lipophilicity and pharmacokinetic properties of the molecule.^[^ ^1^, ^27^ ^]^ Interestingly, in addition to their steric and electronic effects on binding affinity, hydrophobic contacts and hydrogen bonding have often been reported, while no evidence for halogen bonding has not been provided yet.

As regards bithionol, the interest in this compound has risen over the years due to the evidence of its relevant antibacterial,^[^ ^29^ ^]^ anthelmintic, and algaecide^[^ ^30^ ^]^ properties. Moreover, it was found to act as an allosteric inhibitor of intracellular soluble adenylyl cyclase (sAC), an enzyme catalyzing the conversion of adenosine triphosphate to cyclic adenosine monophosphate, a process activated by HCO_3_^–^, one of the products of the CAs‐catalyzed reaction. Interestingly, sAC is also involved in regulating eye pressure and acid–base balance, both of which are also modulated by CAs.^[^ ^31^ ^]^ Notably, the compound was shown to be cytotoxic and photosensitizing in vivo.^[^ ^32^ ^]^

### 2.2 Inhibitory activity against hCAs

As a preliminary step of the study, the ability of bithionol to inhibit a panel of hCAs such as the isoforms I, II, III, and XII was assessed by means of CO_2_ hydration assay through the stopped‐flow technique,^[^ ^33^ ^]^ and the obtained inhibition constant (*K*_I_) values are reported in Table 1.

**Table 1:** Inhibitory data on a panel of hCA isoforms for bithionol.

CPD	*K*_I_ (µM)^a^			
	hCA I	hCA II	hCA III	hCA XII
Bithionol	78.6 ± 5.2	31.2 ± 2.0	>100	92.8 ± 5.4
AAZ	0.25 ± 0.02	0.012 ± 0.001	20.0 ± 1.1	0.006 ± 0.0005

Abbreviations: AAZ, acetazolamide; hCA, human carbonic anhydrase.

^a^ The inhibition constant (*K*_I_) values are expressed as a mean ± SD of three independent experiments by the stopped‐flow technique.^[^ ^33^ ^]^ Errors were in the range of ± 5–10% of the reported values. AAZ was reported as a reference inhibitor.

Observing data reported in Table 1, bithionol displayed micromolar inhibitory activity toward four hCAs, with a clear preference for hCA II (*K*_I_ = 31.2 µM) over hCAs I and XII (*K*_I_ values = 78.6 and 92.8 µM, respectively).

These results are in accordance with previous data that showed simple phenols and terpene essential oils, such as resorcinol, gallic acid, eugenol, vanillin, and flavonoids inhibiting hCAs in the low micromolar range.^[^ ^34^, ^35^, ^36^, ^37^ ^]^

### 2.3 Crystallographic studies

The promising inhibition activity toward hCA II encouraged us to investigate the binding mode of bithionol by solving the X‐ray structure of the hCA II inhibitor complex. Upon refining the structure of the hCA II–bithionol complex to a resolution of 1.35 Å, electron density maps did not reveal the binding of any inhibitor molecule within the catalytic site of the enzyme, neither classically bound to the catalytic zinc ion nor to the neighborhood residues. However, the inhibitor, well‐defined in the electron density (Supporting Information S1: Figure S1), was observed in proximity to the side chain of His64, approximately 10 Å away from the zinc ion. In addition, a second molecule was observed bound to the protein in the α‐helix region between Pro155 and Lys159, almost certainly not implicated in the inhibition process (Supporting Information S1: Figure S1).

**Figure 2:**
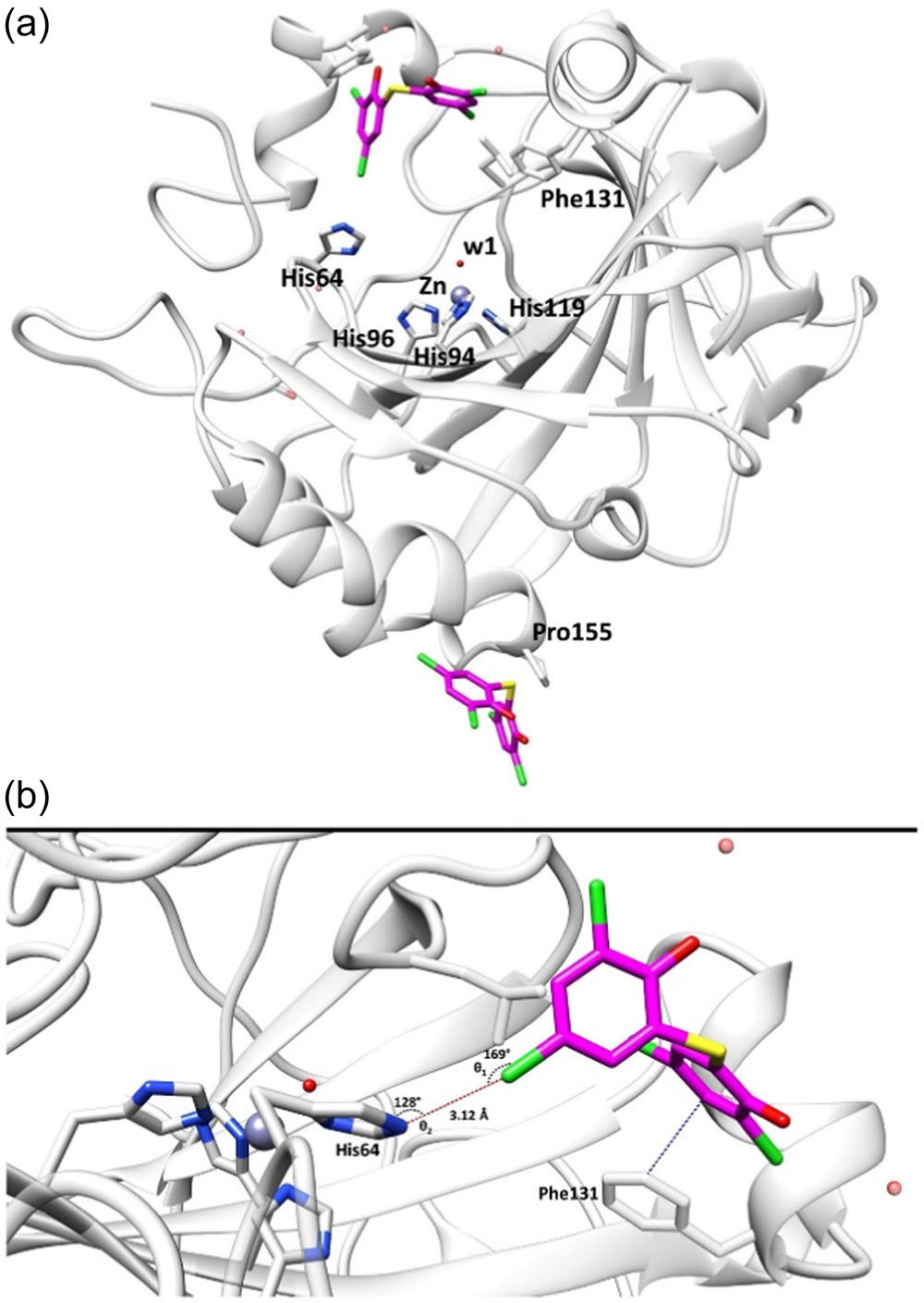
(a) X‐ray crystal structure of hCA II in complex with bithionol, reported in the Protein Data Bank (PDB) with ID: 8QQA; (b) Zoom on chlorine‐nitrogen X‐bond between bithionol and His64. Bithionol is represented by purple sticks and hCA II by a gray cartoon. The interacting residues are represented by gray sticks and labeled. Water molecules and zinc ions are shown as red and blue spheres, respectively. X‐bond (Cl–N) is represented by a red dashed line. Binding angles and distances are also reported.

A focused exploration of the enzyme interactions with the bithionol molecule responsible for the inhibition mechanism (Figure 2a) revealed a highly directional interaction with Nε of His64 by the chlorine atom, characteristic of an X‐bond. The distance of this interaction is 3.12 Å (Figure 2b), closely near to the sum of van der Waals radii (3.08 Å for chlorine and nitrogen), and only slightly longer than the ideal X‐bond length.^[^ ^8^, ^10^ ^]^ This result is in excellent agreement with the value of 3.14 Å reported by Lange et al.^[^ ^38^ ^]^ who investigated the X‐bond between chlorobenzene and two histidine derivatives (capped histidine and imidazole) at a high level of theory. The angles θ1 and θ2 (169° and 128°, respectively, Figure 2b) also agree well with the values computed for the capped histidine (167°) and imidazole (134°).^[^ ^38^ ^]^

### 2.4 In silico calculations

**Figure 3:**
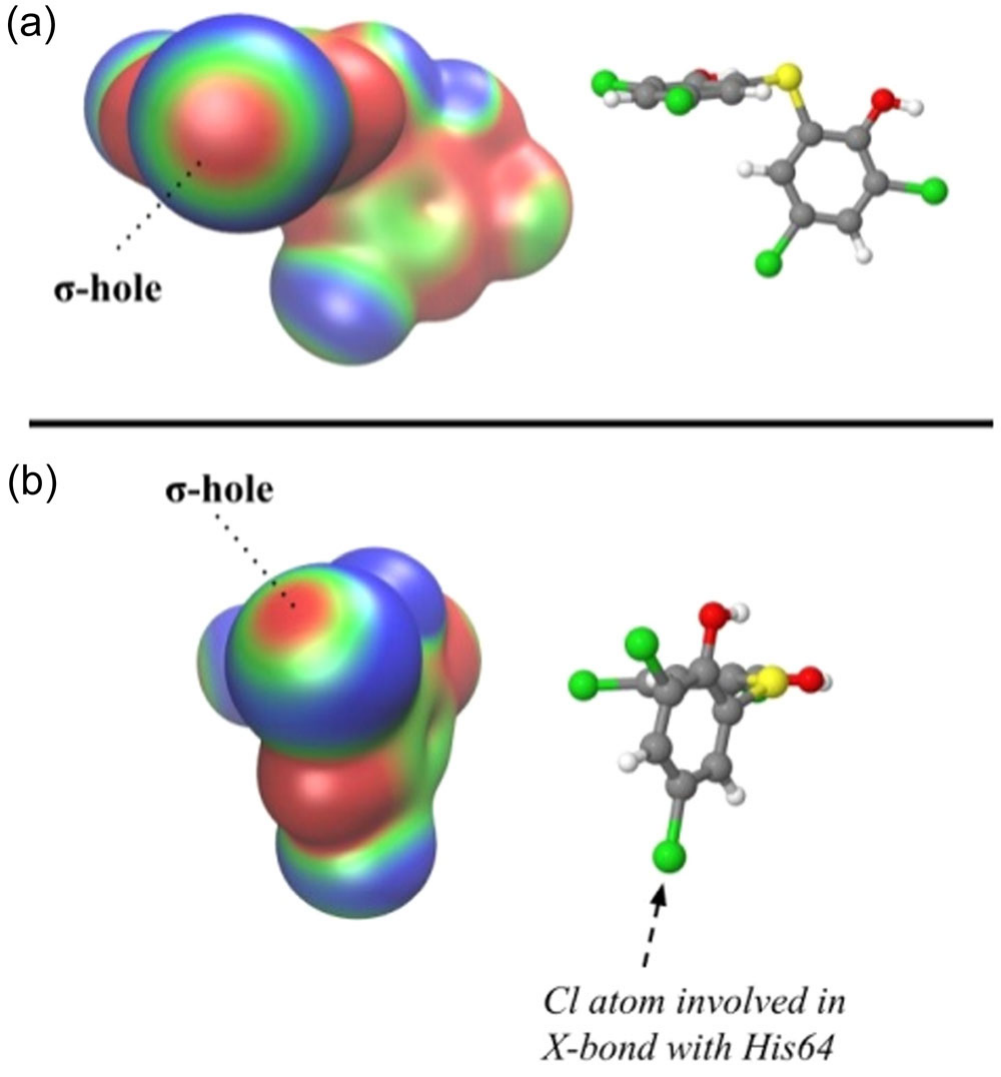
Computed molecular electrostatic potentials (MEPs) at the M06‐2X/jun‐cc‐pVTZ level of theory on the 0.001 electrons/bohr^3^ surface for bithionol oriented as indicated by the corresponding three‐dimensional (3D) structures to show the σ‐hole on the Cl atoms interacting with (a) His64 and (b) Phe20 side chain. Different colors indicate different electronegative regions where blue and red are the most negative and positive regions, respectively.

Molecular electrostatic potential (MEP) calculations allow us to identify and visualize the σ‐hole in halogen atoms,^[^ ^9^, ^39^ ^]^ and its extension can be related to the strength of this intermolecular interaction. MEPs of bithionol were computed at M06‐2X/jun‐cc‐pVTZ level of theory using the X‐ray coordinates and, as shown in Figure 3a, the Cl atom pointing toward the *N3* atom of His64 is clearly characterized by the presence of the σ‐hole.

Examining the geometry of the X‐bond, particularly the angle of approach of the halogen toward the acceptor atom (θ2) (Figure 2b), it can be observed that the σ‐hole is mainly attracted to the nonbonding electrons of the acceptor, exhibiting an angle θ2 of 128°. This finding aligns with the geometries observed in small molecule structures.^[^ ^40^ ^]^ For the angle θ1, a distinct preference for a nearly linear approach of the acceptor toward the electropositive corona of the σ‐hole is typical for this type of bond. However, the increase in the available surface area of the halogen atom often induces a rearrangement of the bond angle, which can vary in a 20° range. This phenomenon explains the angle θ1 of 169° (Figure 2b) observed between the chlorine and nitrogen atoms, a result consistent with previous observations for similar interactions with other nitrogen derivatives.^[^ ^10^, ^40^ ^]^

**Figure 4:**
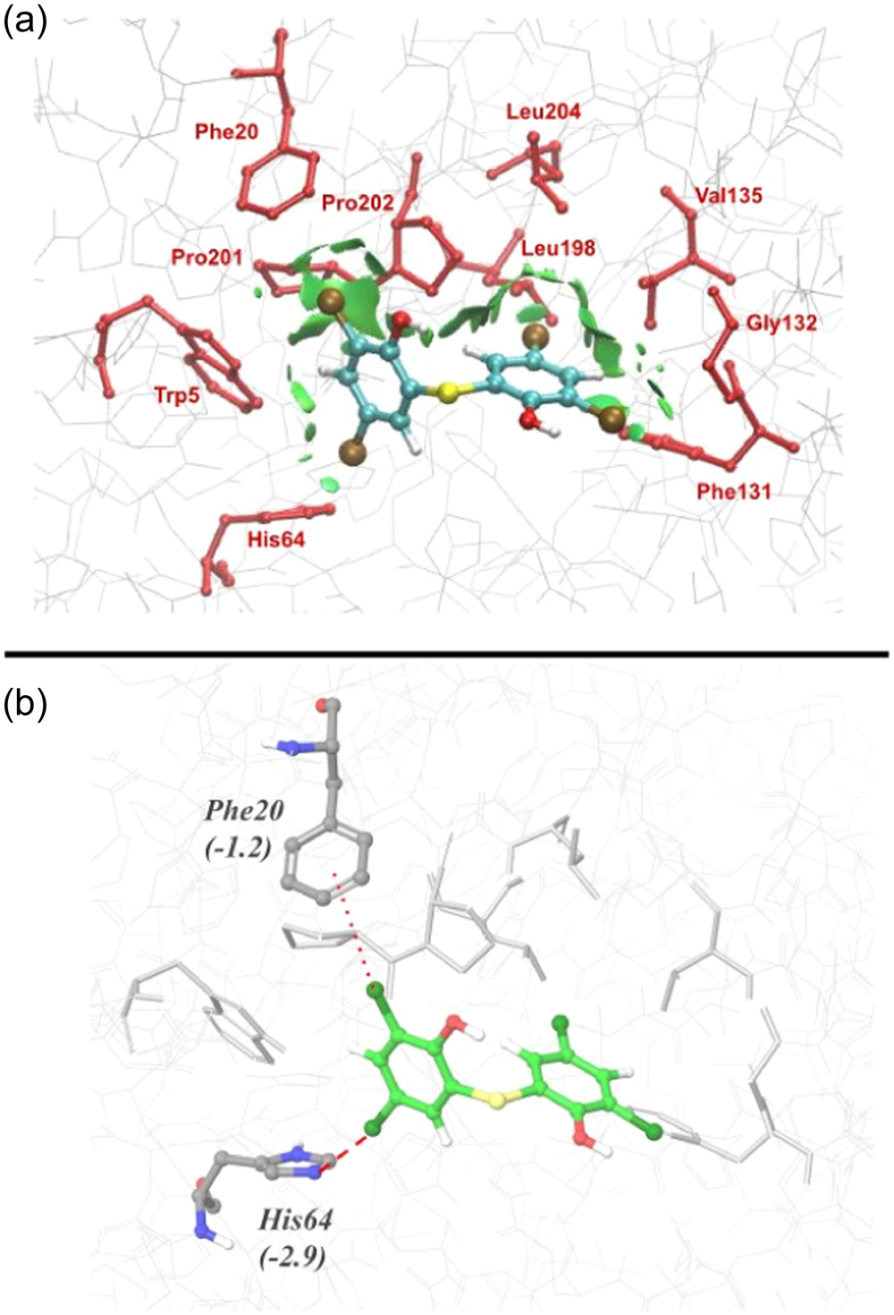
(a) Interaction surface around the density critical point describing the noncovalent interactions (NCIs) between bithionol (in cyan sticks) and residues (in red sticks and labeled) of human carbonic anhydrase (hCA) II within a range of 4 Å; (b) bithionol (in green sticks) and residues of hCA II (PDB ID: 8QQA) within a range of 4 Å (in gray sticks and labeled), used as input structure for fragment molecular orbital (FMO) calculations. The X‐bond involving His64 and Phe20 is represented by a thick and thin red dotted line, respectively. The pair interaction energies (PIEs) of the X‐bond are reported in parenthesis in kcal/mol.

Starting from the refined structure of the X‐ray complex, the noncovalent interaction (NCI) analysis^[^ ^41^ ^]^ was used to assess the nature of the interactions between bithionol and residues lying within a 4 Å distance. As shown in Figure 4a, bithionol interacts with all the surrounding residues by means of moderate or weak contacts as indicated by the green interaction surface.

This evidence agrees with the moderate magnitude of the experimental *K*_I_ value (= 31.2 µM, Table 1) and with the features of the binding pocket since, except for His64, all the other residues are characterized by hydrophobic side chains, such as Trp5, Phe20, Leu198, Pro201, Pro202, Leu204, Val135, Gly132, and Phe131, allowing only dispersion interactions with bithionol.

To assess the strength of X‐bond between His64 and bithionol into the binding pocket, we performed a single‐point calculation with the ab initio fragment molecular orbital (FMO) method^[^ ^42^, ^43^ ^]^ at M06‐2X/6‐31 G(d) level of theory considering bithionol and residues within a range of 4 Å (Trp5, Phe20, His64, Phe131, Gly132, Val135, Leu198, Pro201, Pro202 and Leu204) (Supporting Information S1: Figure S3). Before using the FMO method to characterize the X‐bond in the hCA II‐bithionol complex, the accuracy of this computational approach was verified as described in the section “4.4 Computational study.”

In Supporting Information S1: Table S1 we reported the pair interaction energy (PIE) values, that is, the interaction energies computed between each fragment pair, between bithionol and all residues of the binding pocket (4 Å): the PIE related to His64‐bithionol is –2.9 kcal/mol associated with a charge transfer of 0.0248 (elementary charge unit) from His64 to bithionol indicating that the halogen atom acts as an electron acceptor, as expected for the X‐bond interaction.^[^ ^44^ ^]^

In addition, a PIE value of –1.2 kcal/mol suggests that Phe20‐bithionol interaction has also the features of an X‐bond interaction, though less strong (Figure 4b). Indeed, the bithionol Cl in position 5 is characterized by the presence of a σ‐hole (Figure 3b) and could establish an X‐bond with the π‐electrons of the Phe20 side chain. However, steric clashes with Trp5 forbid reaching the optimal spatial orientation of the Cl‧‧‧Ph bond^[^ ^8^, ^45^, ^46^ ^]^ limiting the strength of this interaction. It is worth noting that this halogen bond could in principle be strengthened by the formation of a hydrogen bond between Cl and the H atom of the adjacent OH group (rotated by180°)^[^ ^47^ ^]^ leading to the so‐called “hydrogen bond enhanced halogen bonds” (HBeXB).^[^ ^48^ ^]^ Nevertheless, FMO calculations showed that the Cl‧‧‧HO hydrogen bond determines a slight reduction of Phe20‐bithionol X‐bond by 0.2 kcal/mol, presumably due to the repulsive interactions between the H atoms of the OH function and the Phe20 side chain.

From the analysis of the X‐ray structure, it emerges that bithionol is surrounded by several nonpolar residues’ side chains suggesting that hydrophobic interaction can play a significant role in LR stabilization. This type of interaction can be estimated by computing the dispersion energy (*E* ^DISP^) contribution by performing the PIE decomposition analysis (PIEDA) implemented in the FMO method.^[^ ^49^ ^]^ We performed an FMO calculation at the RI‐MP2 level of theory which allows calculating the dispersion energy, using the same basis set of DFT analysis and including the solvent effect (PCM[1]). As reported in Supporting Information S1: Table S2, *E* ^DISP^ does represent the largest attractive energy term with 59% of the total attractive energy, confirming that the hydrophobic contacts are dominant in the LR interactions along with X‐bonds.

In fact, another FMO notable result is that bithionol interacts with the binding pocket residues by establishing two X‐bonds (His64 and Phe20) through one phenolic ring whereas the other ring is only involved in the hydrophobic contacts (Figure 4a).

**Figure 5:**
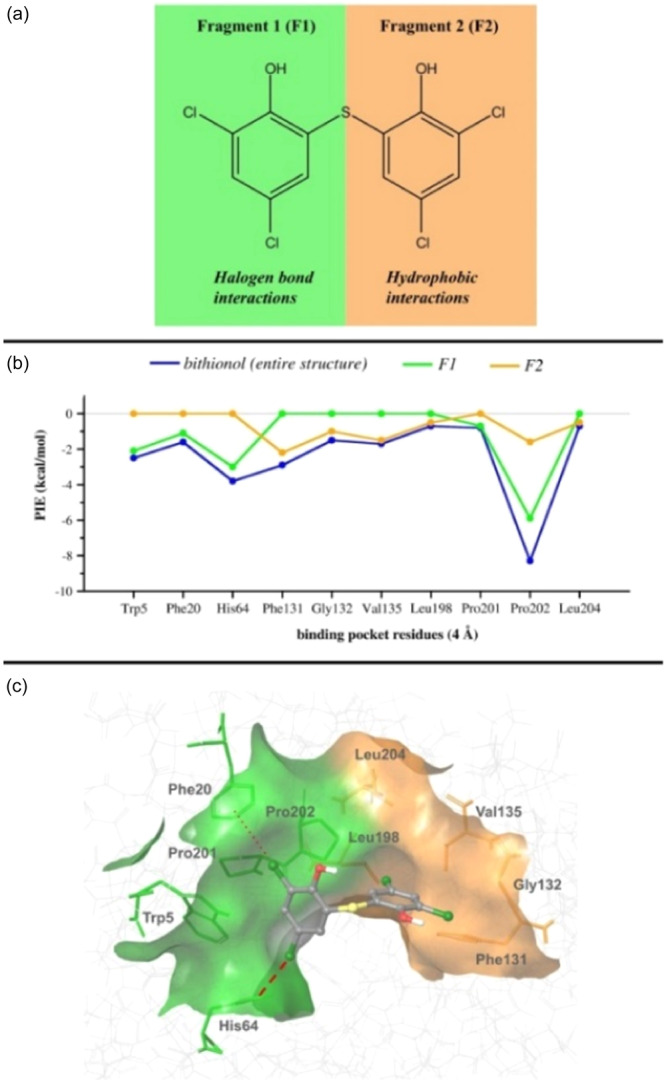
(a) Bithionol split according to its binding properties: one phenolic ring (**F1**, green) is involved in X‐bonds while the second one (**F2**, orange) only establishes hydrophobic interactions; (b) graph of PIEs computed for bithionol, **F1** and **F2** in blue, green and orange lines, respectively, computed at the fragment molecular orbital (FMO) RI‐MP2/6‐31 G(d)//PCM[1] level of theory. All PIE values along with PIEDA are reported in Supporting Information S1: Tables S3 and S4 for **F1** and **F2**, respectively; (c) bithionol and residues within a range of 4 Å, colored according to the interacting ligand fragments (green: X‐bond interactions involving **F1**; orange: hydrophobic contacts involving **F2**). The binding pocket surface is also shown. The X‐bonds with His64 and Phe20 are reported by using thick and thin red dotted lines, respectively.

This issue was further addressed by splitting bithionol into two fragments, **F1** and **F2** (Figure 5a and Supporting Information S1: Figure S4), each one containing a 5,3‐dichloro‐2‐hydroxy‐thiophenol moiety and performing two additional FMO calculations at RI‐MP2/6‐31 G(d)//PCM[1] level of theory.

As shown in Figure 5b, the analysis of PIEs computed for ligand fragments shows that **F1**, placed in the small pocket delimited by Pro201, Tpr5, Phe20, and His64 (green surface in Figure 5c), is mainly involved in two X‐bonds with His64 and, more weakly, with Phe20. On the contrary, **F2** is inserted in a deeper cavity defined by Leu198, Leu204, Val135, Gly132, and Phe131, establishing only hydrophobic interactions (orange surface in Figure 5b,c). Indeed, Leu198 borders the two regions of the binding site interacting with both ligand fragments, however, the strongest interaction is computed with **F1** (Supporting Information S1: Tables S3 and S4).

These results provide a detailed description of the chemical features of the binding pocket targeted by bithionol and suggest a possible strategy to design new and stronger bithionol derivatives. Considering that the binding site is formed by two distinct regions, one able to accept X‐bonds and the other to establish hydrophobic interactions, a new binder should be an asymmetric bithionol derivative with one portion (**F1**, containing one or two halogen atoms, such as Cl, Br, or I) optimized to establish strong X‐bonds with His64 (and with Phe20) and the other one (**F2**) specifically designed to maximize the interactions with the hydrophobic pocket delimited by Leu198, Leu204, Val135, Gly132, and Phe131.

So far crystallographic data and quantum mechanical calculations converge to highlight the existence of X‐bonding in the bithionol‐hCA II complex and that it is the key factor for the binding of one of its phenolic rings, likely enhancing its inhibitory activity. An independent indirect corroboration of the effect of X‐bonding in modulating hCA inhibition comes again from the inhibitory data reported in Table 1. Bithionol is inactive against hCA III notwithstanding its high sequence homology with hCA II: In fact, hCA III features the crucial substitution of the proton shuttle His64 with Lys64, which is positively charged at physiological pH, forbidding the formation of an X‐bond. In addition, hCA III is characterized by a different hydrophobic active site due to the mutation of Leu198Phe, which sterically hinders the adaptation of the **F2** moiety in the pocket delimited by Gly132, Phe131, Leu198, Pro202, Leu204, and Val135, possibly reducing the strength of hydrophobic interactions (Figure 5c).

## 3 CONCLUSIONS

In conclusion, the present study uncovers a distinctive and unprecedented binding mode of halogenated phenols, exemplified by the case study of bithionol within the hCA II active site. In particular, bithionol interacts with the catalytically critical His64 residue, which plays a key role in the rate‐limiting step of the enzyme catalytic activity, via a halogen‐bonding interaction. Interestingly, the compound establishes an additional interaction of the same type, but weaker, with the Phe20 residue. Overall, these interactions were elucidated by detailed structural analysis using X‐ray crystallography, complemented by quantum chemical calculations, providing a remarkable insight into the molecular interactions involved at the atomic level.

Apart from being unheard of in the field, our findings could represent a significant advance in the understanding of the mechanistic of the binding mode and consequent affinity of halogenated compounds to the hCA II active site. However, several challenges remain, such as the translation of this achievement into the rational design of CA modulators fully exploitable, and a wide range of investigative opportunities are now open.

Although bithionol represented a compelling case study for our research, enzymatic data showed the compound to be a weak hCA II inhibitor with a low selective profile over the other tested CAs, suggesting that, to be susceptible to inhibition, the CA enzyme seems to only require the presence of the histidine proton shuttle residue. Therefore, bithionol inhibitory potency and selectivity need to be improved by chemical modification, with special focus paid to those that allow the enhancement of the halogen bonding interaction. With this aim, a series of bithionol derivatives, and, in general, halogenated phenols, will be designed and synthesized through a continuous feedback interactive process based on derivative preparation and functional activity evaluation.

Moreover, an in‐depth study is needed to explore the full potential of halogen‐bonding interactions of compounds containing halogen atoms other than chlorine within hCA II. Also, the key contribution of this interaction to the enzyme‐inhibitor complex requires further investigation across the different (h)CA isoforms. In line with this, future steps in this research will focus on expanding the profiling of the CAs inhibitory activity of halogenated phenols. A broad panel of halogenated compounds will be tested using a validated combination of enzymatic assays, X‐ray crystallography, and quantum chemical calculations to obtain kinetic and structural characterization.

However, another crucial challenge lies in the presence of halogen atoms in the compounds. Indeed, although the optimization process could lead to the introduction of halogens into the chemical structures of known CA inhibitors to implement their affinity and inhibitory potency, their potential off‐target mechanisms should not be overlooked. Although known to enhance drug‐like properties, the introduction of one or more halogens in active compounds is reported to increase their reactivity, which results in causing oxidative stress and exhibiting in vivo toxicity, including nephron and hepato‐toxicity and endocrine disruption. The persistence of halogens in the environment may also pose additional risks. To avoid the occurrence of side effects, the library of halogenated compounds should be designed rationally, for example, by molecular modeling, while predicting their safety profile using predictive tools. However, several halogenated drugs have been approved in the last decades, confirming the possibility of successfully balancing beneficial effects and safety in the compound of interest.

In summary, this innovative binding mode could pave the way for the design of more potent and selective CA inhibitors, including those specifically targeting the histidine proton shuttle residue.

## 4 EXPERIMENTAL

### 4.1 CA inhibition assays

An Applied Photophysics stopped‐flow instrument was used to evaluate the ability of the test compounds to inhibit the CA‐catalyzed CO_2_ hydration.^[^ ^33^ ^]^ Phenol red (at a concentration of 0.2 mM) was chosen as an indicator (absorbance maximum = 557 nm), with 20 mM HEPES (pH 7.4 for α‐CAs) as a buffer, and 20 mM Na_2_SO_4_ (to maintain constant the ionic strength), following the initial rates of the CA‐catalyzed CO_2_ hydration reaction for a period of 10–100 s. The CO_2_ concentrations ranged from 1.7 to 17 mM for the determination of the kinetic parameters and inhibition constants. The enzyme concentrations in the assay system were as follows: hCA I, 13.2 nM; hCA II, 8.4 nM; hCA III, 17.0 nM; hCA XII, 15.2 nM. Stock solutions of the inhibitor (0.1 mM) were prepared in distilled–deionized water and dilutions up to 0.01 nM were prepared with the assay buffer. Inhibitor and enzyme solutions were preincubated for 15 min at r.t. for the formation of the E–I complex. The inhibition constants were obtained by nonlinear least‐squares methods using PRISM 3 and the Cheng–Prusoff equation^[^ ^50^ ^]^ and represent the mean from at least three different determinations. Apart from human CAs I and II purchased from Merck, all other CAs (hCA III and hCA XII) are recombinant and obtained *in house*.^[^ ^51^, ^52^ ^]^

### 4.2 Crystallization and X‐ray data collection

Crystals were obtained using the hanging drop vapor diffusion method using 24 well Linbro plate. 2 µL of 10 mg/mL solution of hCA II in Tris‐HCl pH 8.0 was mixed with a solution of 1.5 M sodium citrate, 50 mM Tris pH 8.0, and were equilibrated against 500 µL of the same solution at 296 K. Crystals of the complexes grew in a few days. hCA II crystals were soaked in 5 mM inhibitor solution for 1 day. The crystals were flash‐frozen at 100 K using a solution obtained by adding 25% (*v/v*) glycerol to the mother liquor solution as a cryoprotectant. Data on crystals of the complexes were collected using synchrotron radiation at the XRD2 beamline at Elettra Synchrotron (Trieste, Italy) with a wavelength of 1.00 Å and a DECTRIS Pilatus 6 M detector. Data were integrated and scaled using the program XDS.^[^ ^53^ ^]^ Data processing statistics are in Supporting Information S1: Table S5.

### 4.3 Structure determination

The crystal structure of hCA II (PDB accession code: 4FIK)^[^ ^54^ ^]^ without solvent molecules and other heteroatoms was used to obtain the initial phases of the structures using Refmac5.^[^ ^55^ ^]^ A total of 5% of the unique reflections were selected randomly and excluded from the refinement data set for the purpose of Rfree calculations. The initial |Fo–Fc| difference electron density maps unambiguously showed the inhibitor molecules. Refinements proceeded using normal protocols of positional, isotropic atomic displacement parameters alternating with the manual building of the models using COOT.^[^ ^56^ ^]^ The quality of the final models was assessed with COOT and RAMPAGE.^[^ ^57^ ^]^ Atomic coordinates were deposited in the Protein Data Bank (PDB accession code: 8QQA). Graphical representations were generated through Chimera.^[^ ^58^ ^]^

### 4.4 Computational study

The interaction between bithionol and hCA II binding site was investigated by using several computational approaches paying special attention to the characterization of the X‐bond established with His64 side chain. First, the X‐ray structure of the hCA II‐bithionol complex (PDB ID: 8QQA) was refined by using the CHRMM‐GUI (*e.g*., adding H atoms, fixing the protonation states of the side chain)^[^ ^59^, ^60^, ^61^ ^]^ and then used to carry out a first evaluation of LR interactions by the NCI analysis,^[^ ^41^ ^]^ considering only the ligand and residues within a range of 4 Å. In the NCI approach, the features of the halogen bond can be investigated by analyzing the sign of the second derivative of the electronic density in the perpendicular direction of the bond (λ_2_), at a critical point where the reduced gradient is equal to zero, times the density (ρ) leading to sign (λ_2_)·ρ term. A negative or a positive value of λ_2_ indicates an attractive interaction or a steric repulsion, respectively, while a value very close to zero indicates weak van der Waals interactions.^[^ ^9^ ^]^ NCI calculations were performed using the NCI‐plot software.^[^ ^62^, ^63^, ^64^ ^]^

The MEP was computed at M06‐2X/jun‐cc‐pVTZ to characterize the σ‐hole on the halogen atoms of bithionol. This level of theory was selected since the M06‐2X hybrid functional, coupled with the Dunning augmented triple‐basis set (e.g., aug‐cc‐pVTZ), was shown to accurately describe the geometries and energies of halogen bond interactions.^[^ ^9^, ^65^, ^66^ ^]^ In this study, the calendar jun‐cc‐pVTZ basis set^[^ ^67^ ^]^ was adopted instead of aug‐cc‐pVTZ because it provides good reliability with reduced computational cost.

Finally, to estimate the X‐bond interaction energy of bithionol in the binding pocket we performed a single‐point calculation using the FMO method^[^ ^42^, ^43^ ^]^ at M062X/6‐31 G(d) level of theory considering the ligand and all the residues within a distance of 4 Å.

Within the FMO frame, the system can be split into several fragments (e.g., one fragment for each amino acid of the protein and ligand) and the total energy of the system can be computed as the sum of the internal energy of each fragment and the so‐called PIEs, that is the interaction energies computed between each fragments pair. The considered protein structure was split into fragments, each one containing a single amino acid, and the covalent bond connecting Cα and the NH group was selected as the fragmentation point, using the hybrid orbital projection (HOP) treatment for bond detachment.^[^ ^68^ ^]^ The amino acid termini resulting after backbone cutting, –NH and –C═O, were capped by adding H atoms.^[^ ^69^ ^]^

PIE can be examined by performing the decomposition analysis (PIEDA)^[^ ^70^, ^71^ ^]^ providing important information on the nature of chemical interaction between fragment pairs (Equation 2):

(1) $$\text{PIE}=E^{\text{ES}}+E^{\text{EX}}+E^{\text{CT}}+E^{\text{DISP}}+E^{\text{SOLV}},$$

where *E* ^ES^, *E* ^EX^, *E* ^CT^, E^DISP^, and *E* ^SOLV^ are the electrostatic, exchange, charge transfer, dispersion, and solvation energy, respectively. In Equation (1), if a DFT method is used, *E* ^DISP^ is replaced by *E* ^RC^ which is the energy term related to the remainder correlation.^[^ ^72^ ^]^ *E* ^SOLV^ is present in PIEDA only if an implicit solvation method is included in the calculations.

The sum of all PIEs of a ligand (L) with all other residues (i) represents the total interaction energy, *E* ^INT^, and provides an estimation of the binding strength of the LR complex frozen in the binding conformation (Equation 3):

(2) $$E^{\mathrm{INT}}=\sum {\text{PIE}}_{Li}.$$

Although *E* ^INT^ does not represent a real binding energy,^[^ ^43^, ^73^ ^]^ it can be used to estimate the LR binding affinity providing generally a good correlation with experimental binding data.^[^ ^74^, ^75^ ^]^ The consistency of the FMO method to evaluate X‐bond strength was assessed by comparing the X‐bond energy, *E* ^XB^, with PIE values computed for a specific set of noncovalent complexes characterized by the Cl‧‧‧N halogen bond shown in Supporting Information S1: Figure S5. In detail, we considered the noncovalent complexes formed by 5‐methylimidazole (MIM), as mimetic of His side chain, and 2‐(3,5‐dichlorophenol)thio‐ethylene (**1**) together with 12 derivatives, as bithionol analogs, where the phenolic group is not involved in the X‐bond has been replaced by an ethenyl moiety to reduce the number of atoms. Geometry optimization calculations at M06‐2X/6‐311++G(d,p) level of theory were performed for the noncovalent complexes and for the isolated forms of MIM and bithionol analogs (**1–13**). The energy of the optimized structures was then refined by performing a single point calculation at M06‐2X/jun‐cc‐pVTZ level of theory and *E* ^XB^ was computed using the following formula (Equation 3):

(3) $$E^{\text{XB}}=E\left(\text{MIM}-1\right)-\left[E\left(1\right)+E\left(\text{MIM}\right)\right].$$

As shown in Supporting Information S1: Figure S6, the PIE values are in good agreement with *E* ^XB^ (*R* ^2^ = 0.916), suggesting that the FMO method can be a valuable computational tool for assessing the X‐bond in biological systems. An RMSE of 0.3 kcal/mol indicates that PIE, computed at M06‐2X/6‐31 G(d) level of theory, slightly overestimates the X‐bond strength (Supporting Information S1: Table S6).

Considering that the dispersion energy can provide an estimation of the hydrophobic interaction which plays an important role in the hCA II‐bithionol interaction, we also performed FMO calculations at RI‐MP2/6‐31 G(d) using the PCM[1] method^[^ ^76^ ^]^ coupled with the partial screening method^[^ ^77^ ^]^ to simulate the solvent effect.

The same level of theory was adopted to assess separately the contribution to the binding process of different scaffolds of the ligand^[^ ^69^, ^74^ ^]^ and the two phenolic rings of bithionol were split into two distinct fragments, **F1** and **F2**, as schematized in Supporting Information S1: Figure S4. The S atom resulting from the detached bond is saturated by adding an H atom. The consistency of this approach was assessed by comparing the PIEs computed for bithionol with the sum of PIEs determined for **F1** and **F2** showing a high correlation (*R* ^2^ = 0.994).

All FMO calculations were performed by using the GAMESS‐US package (version: 30 June 2021—R1)^[^ ^78^ ^]^ while MEPs and DFT calculations for the *E* ^XB^ evaluation were performed by using Gaussian09 package.^[^ ^79^ ^]^

## CONFLICT OF INTEREST STATEMENT

The authors declare no conflicts of interest.

## Supporting information

Supplementary Information

## Data Availability

The data that support the findings of this study are available from the corresponding author upon reasonable request.
