# Supplementary material for: Unprecedented carbonic anhydrase inhibition mechanism: Targeting histidine 64 side chain through a halogen bond
Source: Arch Pharm (Weinheim). 2025 Jan 6;358(1):e2400776. doi: 10.1002/ardp.202400776 (PMC11704030; doi:10.1002/ardp.202400776)
Supplement: Supplementary file 1 — Supplementary Information [file ARDP-358-e2400776-s001.pdf]

SUPPORTING INFORMATION

Unprecedented Carbonic Anhydrase Inhibition Mechanism: Targeting Histidine 64 Side Chain Through a Halogen Bond.

Roberto Paciotti,[1,‡] Simone Carradori,[1,‡] Andrea Angeli,[2,*] Ilaria D'Agostino,[3] Marta Ferraroni,[4] Cecilia Coletti,[1] and Claudiu T. Supuran.[2]

[1]Department of Pharmacy, "G. d'Annunzio" University of Chieti-Pescara, via dei Vestini, 33, 66100, Chieti, Italy

[2]Section of Pharmaceutical and Nutraceutical Sciences, Department of Neuroscience, Psychology, Drug Research and Child Health (NEUROFARBA), University of Florence, via Ugo Schiff 6, 50019 Sesto Fiorentino, Firenze, Italy

[3]Department of Pharmacy, University of Pisa, via Bonanno Pisano 6, 56126, Pisa, Italy

[4]Department of Chemistry "Ugo Schiff", University of Florence, via della Lastruccia 3, 50019, Sesto Fiorentino, Florence, Italy

[‡]These authors contributed equally to the paper

List of Contents

In silico studies 2

Figure S1. (A) Electron density of bithionol bound Histidine 64 in hCA II active site. (B) Electron density of bithionol bound lysine 159 and proline 155 out of hCA II active site. 2

Figure S2. Bithionol complex with hCA II not involved in the inhibition mechanism. 2

Figure S3. Bithionol (in green sticks) and residues (in gray sticks and labelled) within a range of 4 Å used as input structures for FMO calculations. 3

Figure S4. (A) Fragmentation scheme used to split bithionol in two fragments, F1 and F2. The S atom was retained in both fragments and saturated by adding an H atom; (B) two single-point FMO calculations were performed where F1 and F2 maintained the same coordinates of the corresponding portions in the entire ligand. 3

Table S1. PIEs and the corresponding decomposition analysis computed for bithionol and the binding pocket residues (4 Å) at M06-2X/6-31G(d) level of theory. 4

Table S2. PIEs and the corresponding decomposition analysis computed for bithionol and the binding pocket residues (4 Å) at RI-MP2/6-31G(d)//PCM[1] level of theory. 4

Table S3. PIEs and the corresponding decomposition analysis computed for F1 and the binding pocket residues (4 Å) at RI-MP2/6-31G(d)//PCM[1] level of theory. 5

Table S4. PIEs and the corresponding decomposition analysis computed for F2 and the binding pocket residues (4 Å) at RI-MP2/6-31G(d)//PCM[1] level of theory. 5

Crystallography studies 6

Table S5. Summary of data collection and atomic model refinement statistics for hCA II. 6

Inhibition curves 7

Expertimental section 8

Figure S5. 2D structure of the non-covalent complexes characterized by the presence of Cl-bond and used to validate the consistency of the FMO results. 8

Figure S6. Correlations between E^{XB} and PIE values computed for the non-covalent complexes characterized by the presence of Cl-bond. 8

Table S6. X-bond energy (E^{XB}) and pair interaction energies (PIEs) computed for the non-covalent complexes characterized by the presence of Cl-bond. 9

In silico studies

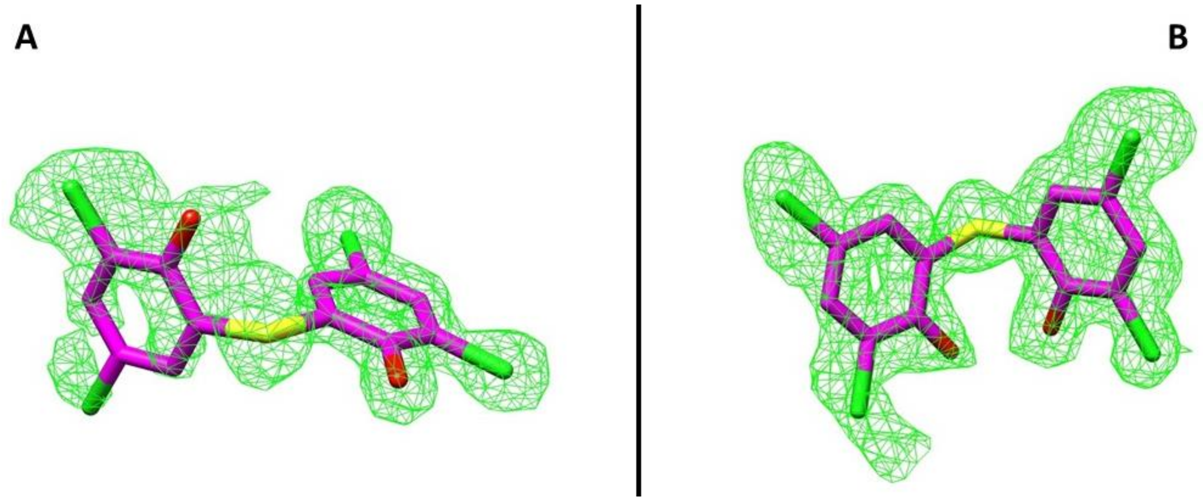

Figure S1. (**A**) Electron density of bithionol bound Histidine 64 in hCA II active site. (**B**) Electron density of bithionol bound lysine 159 and proline 155 out of hCA II active site.
$2F_o$-F_c maps and contoured to the 1.0 σ level.

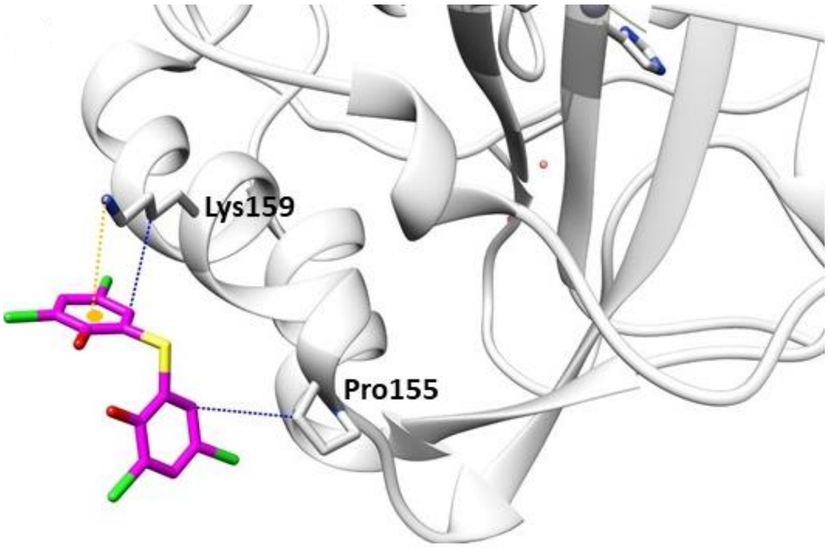

Figure S2. Bithionol complex with hCA II not involved in the inhibition mechanism.

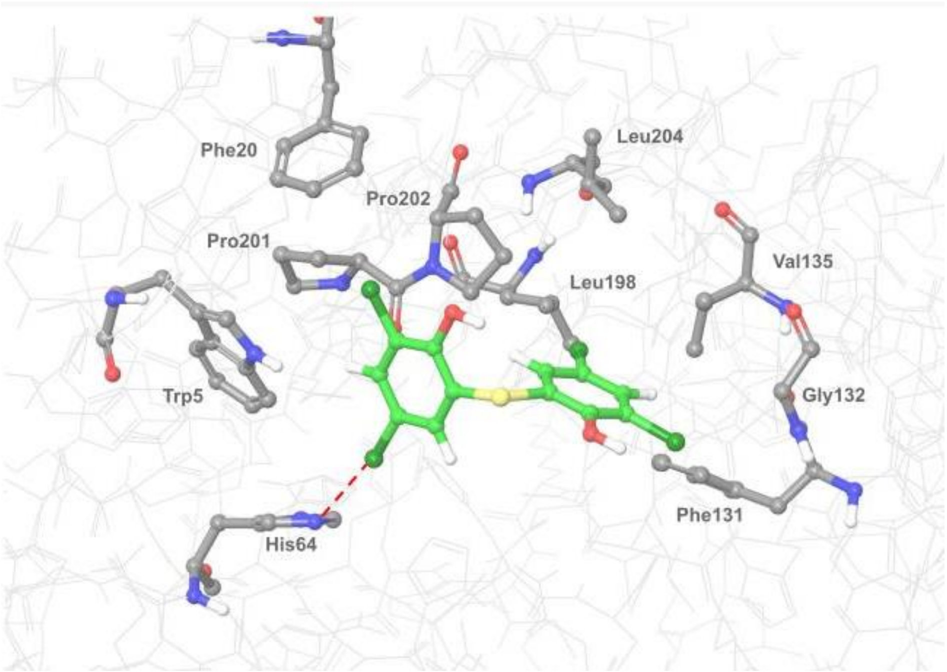

Figure S3. Bithionol (in green sticks) and residues (in gray sticks and labelled) within a range of 4 Å used as input structures for FMO calculations.
The X-bond involving His64 is represented by a red dotted line.

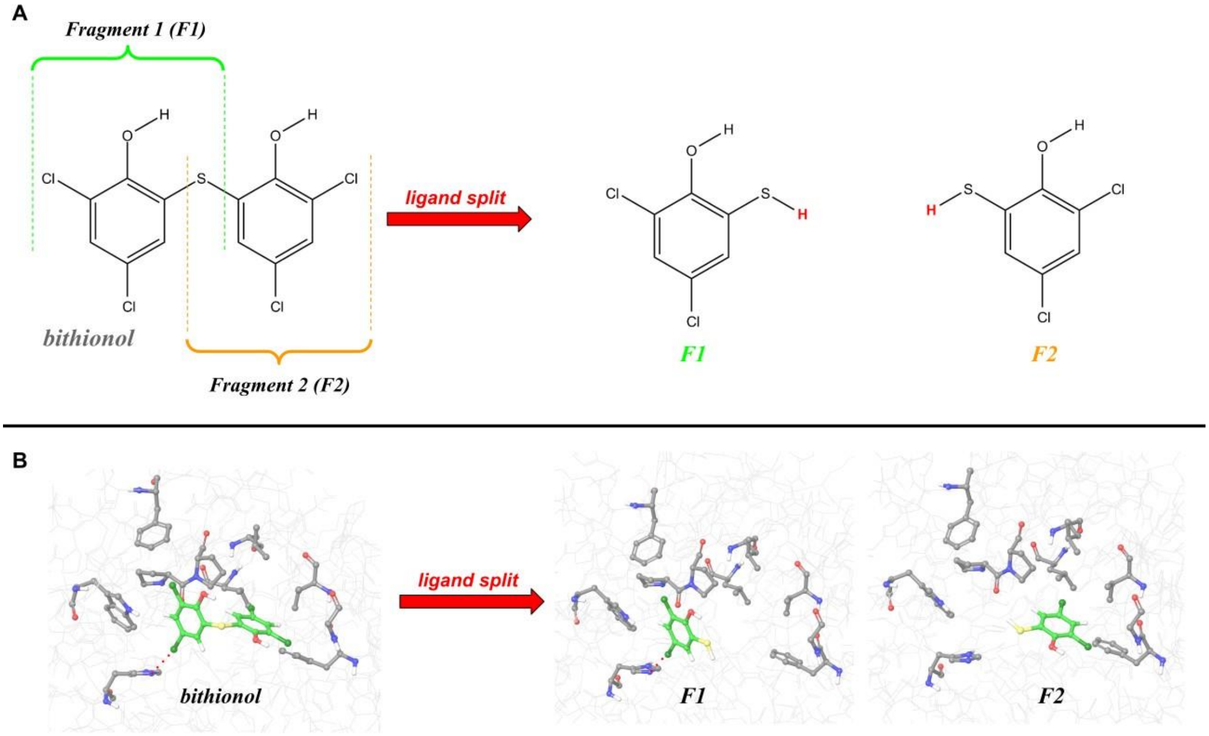

Figure S4. (**A**) Fragmentation scheme used to split bithionol in two fragments, **F1** and **F2**. The S atom was retained in both fragments and saturated by adding an H atom; (**B**) two single-point FMO calculations were performed where F1 and F2 maintained the same coordinates of the corresponding portions in the entire ligand**.**

Table S1. PIEs and the corresponding decomposition analysis computed for bithionol and the binding pocket residues (4 Å) at M06-2X/6-31G(d) level of theory.
All energy values are in kcal/mol.

Pair interaction with	PIE	PIEDA			
		E^{ES}	E^{EX}	E^{CT}	E^{RC}
Trp5	-1.0	0.1	2.6	-1.2	-2.5
Phe20	-1.2	-0.6	2.8	-0.7	-2.6
His64	-2.9	-2.0	3.5	-1.6	-2.9
Phe131	-1.6	-1.2	5.4	-1.2	-4.6
Gly132	-0.7	-0.4	2.2	-0.5	-2.1
Val135	-1.3	-0.5	4.2	-0.8	-4.2
Leu198	-0.6	-0.3	1.9	-0.4	-1.8
Pro201	-0.6	0.0	1.4	-0.4	-1.6
Pro202	-8.0	-4.0	11.2	-2.7	-12.5
Leu204	-0.4	-0.2	0.5	-0.2	-0.5
**E^{INT}**	-18.2	-9.1	35.8	-9.6	-35.3

Table S2. PIEs and the corresponding decomposition analysis computed for bithionol and the binding pocket residues (4 Å) at RI-MP2/6-31G(d)//PCM[1] level of theory.
All energy values are in kcal/mol.

Pair interaction with	PIE	PIEDA				
		E^{ES}	E^{EX}	E^{CT}	E^{RC}	E^{SOL}
Trp5	-2.5	0.0	1.4	-0.8	-2.8	-0.3
Phe20	-1.6	-0.7	1.6	-0.4	-2.0	-0.1
His64	-3.8	-2.0	2.4	-1.1	-2.5	-0.6
Phe131	-2.9	-1.2	3.3	-0.8	-3.6	-0.6
Gly132	-1.5	-0.3	1.4	-0.3	-1.5	-0.7
Val135	-1.7	-0.3	2.4	-0.4	-2.7	-0.7
Leu198	-0.7	-0.2	1.1	-0.2	-1.2	-0.2
Pro201	-0.8	-0.1	0.8	-0.2	-1.3	-0.1
Pro202	-8.3	-4.8	6.3	-2.0	-8.4	0.5
Leu204	-0.7	-0.2	0.3	-0.1	-0.7	0.1
**E^{INT}**	-24.5	-9.8	21.0	-6.3	-26.7	-2.6

Table S3. PIEs and the corresponding decomposition analysis computed for **F1** and the binding pocket residues (4 Å) at RI-MP2/6-31G(d)//PCM[1] level of theory.
All energy values are in kcal/mol.

Pair interaction	PIE	PIEDA				
		E^{ES}	E^{EX}	E^{CT}	E^{RC}	E^{SOL}
F1-Trp5	-2.1	-0.3	1.4	-0.8	-2.8	0.4
F1-Phe20	-1.1	-0.7	1.6	-0.5	-1.9	0.4
F1-His64	-3.0	-1.9	2.4	-1.1	-2.4	0.0
F1-Phe131	0.0	0.1	0.0	0.0	0.0	0.0
F1-Gly132	0.0	0.3	0.0	0.0	0.0	-0.3
F1-Val135	0.0	0.2	0.0	0.0	0.0	-0.2
F1-Leu198	0.0	0.1	0.0	0.0	0.0	-0.1
F1-Pro201	-0.7	-0.2	0.8	-0.2	-1.2	0.1
F1-Pro202	-5.9	-3.8	4.2	-1.6	-5.9	1.1
F1-Leu204	0.0	-0.2	0.0	0.0	0.0	0.2
E^{INT}	-12.7	-6.3	10.5	-4.1	-14.2	1.5

Table S4. PIEs and the corresponding decomposition analysis computed for **F2** and the binding pocket residues (4 Å) at RI-MP2/6-31G(d)//PCM[1] level of theory.
All energy values are in kcal/mol.

Pair interaction	PIE	PIEDA				
		E^{ES}	E^{EX}	E^{CT}	E^{RC}	E^{SOL}
F2-Trp5	0.0	0.3	0.0	0.0	0.0	-0.3
F2-Phe20	0.0	0.0	0.0	0.0	0.0	0.0
F2-His64	0.0	-0.1	0.0	0.0	0.0	0.1
F2-Phe131	-2.2	-1.2	3.4	-0.8	-3.6	0.1
F2-Gly132	-1.0	-0.5	1.4	-0.3	-1.5	0.0
F2-Val135	-1.5	-0.6	2.4	-0.4	-2.7	-0.2
F2-Leu198	-0.5	-0.4	1.1	-0.2	-1.1	0.1
F2-Pro201	0.0	0.1	0.0	0.0	0.0	-0.1
F2-Pro202	-1.6	-0.5	2.0	-0.4	-2.6	0.0
F2-Leu204	-0.5	0.0	0.3	-0.1	-0.7	0.0
E^{INT}	-7.2	-2.8	10.5	-2.3	-12.2	-0.4

Crystallography studies

Table S5. Summary of data collection and atomic model refinement statistics for hCA II.

	**hCA II + bithionol**
PDB ID	8QQA
Wavelength (Å)	1.00
Space Group	$P2_1$
Unit cell (a, b, c, α, β, γ) (Å,°)	42.38, 41.36, 72.38, 90.00, 104.558, 90.00
Limiting resolution (Å)	70.06-1.35 (1.38-1.35)
Unique reflections	49852 (2329)
Rmerge (%)	7.0 (62.2)
Rmeas (%)	7.7 (71.1)
Redundancy	5.35 (4.1)
Completeness overall (%)	92.2 (58.7)
<I/σ(I)>	13.42 (2.28)
CC (1/2)	99.7 (71.5)
**Refinement statistics**	
Resolution range (Å)	70.06-1.36
Rfactor (%)	11.62
Rfree (%)	16.41
r.m.s.d. bonds (Å)	0.0153
r.m.s.d. angles (°)	1.9406
**Ramachandran statistics** (%)	
Most favored	97.7
additionally allowed	2.3
outlier regions	0.0
**Average B factor (Å^2)**	
All atoms	19.653
Inhibitors	39.611
Solvent	30.683

Inhibition curves

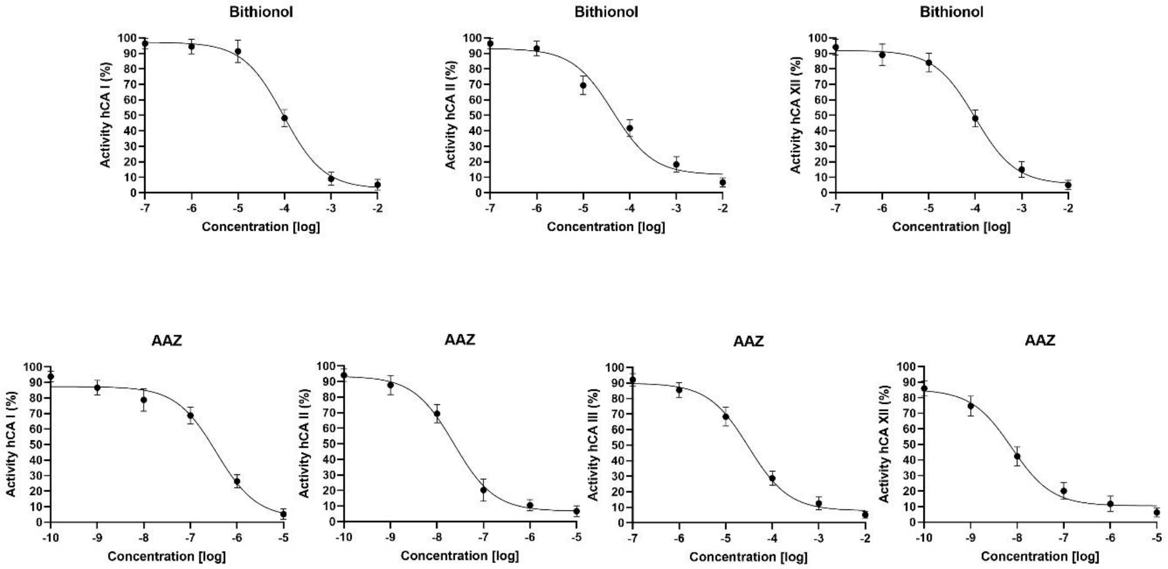

Expertimental section

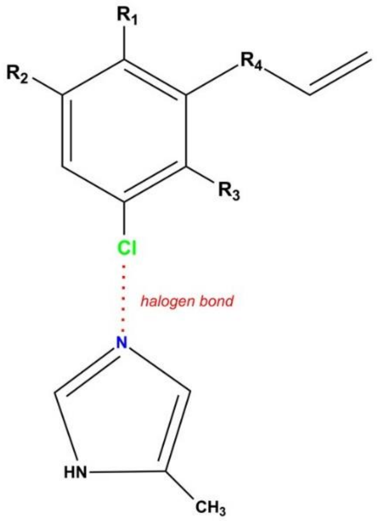

bithionol analogues	R_1	R_2	R_3	R_4
1	OH	Cl	H	S
2	H	Cl	H	S
3	OH	F	H	S
4	OH	Br	H	S
5	OH	I	H	S
6	OH	Cl	H	CH_2
7	OH	Cl	H	O
8	OH	Cl	H	SO_2
9	OCH_3	Cl	H	S
10	OAc	Cl	H	S
11	OH	H	H	S
12	H	H	H	S
13	OH	Cl	Cl	CH_2

Figure S5. 2D structure of the non-covalent complexes characterized by the presence of Cl-bond and used to validate the consistency of the FMO results.
Complexes are formed by 5-methylimidazole (MIM) and 2-(3,5-dichlorophenol)thio-ethylene with its derivatives as mimetic of His side chain and bithionol analogues (**1-13**), respectively.

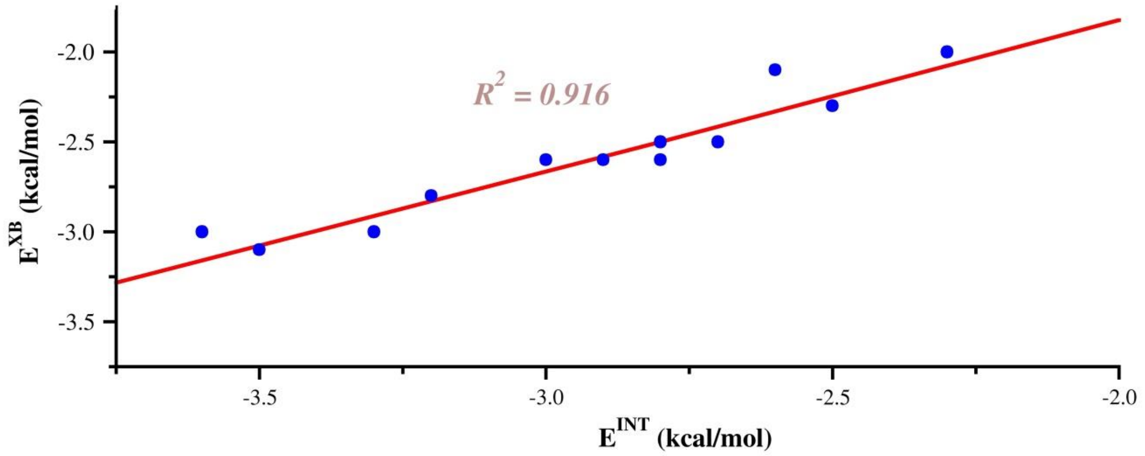

Figure S6. Correlations between E^{XB} and PIE values computed for the non-covalent complexes characterized by the presence of Cl-bond.
The correlation coefficient (R^2) and line (in red) are also shown.

Table S6. X-bond energy (E^{XB}) and pair interaction energies (PIEs) computed for the non-covalent complexes characterized by the presence of Cl-bond.
The deviation values between E^{XB} and PIE are also reported (RMSE = 0.3 Kcal/mol). All energy values are in kcal/mol.

Analogue of bithionol interacting with MIM	E^{XB} [a]	PIE [b]	E^{XB} - PIE
**1**	-2.6	-2.8	0.2
**2**	-2.8	-3.2	0.4
**3**	-2.5	-2.7	0.2
**4**	-2.6	-2.9	0.3
**5**	-2.5	-2.8	0.3
**6**	-2.3	-2.5	0.2
**7**	-2.5	-2.8	0.3
**8**	-3.1	-3.5	0.4
**9**	-2.6	-3.0	0.4
**10**	-3.0	-3.6	0.6
**11**	-2	-2.3	0.3
**12**	-2.1	-2.6	0.5
**13**	-3.0	-3.3	0.3

[a] level of theory: M06-2X/6-311++G(d,p)//jun-cc-pVTZ;
[b] level of theory: FMO M06-2X/6-31(d).
